# Inflammatory Markers Showed Significant Incremental Value for Predicting Post-Hepatectomy Liver Failure in Hepatocellular Carcinoma Patients

**DOI:** 10.3390/life13101990

**Published:** 2023-09-29

**Authors:** Xiao Wang, Wenjun Wang, Xixiang Lin, Xu Chen, Mingxiang Zhu, Hongli Xu, Kunlun He

**Affiliations:** 1 Medical Big Data Research Center, Chinese PLA General Hospital, Beijing 100853, China; wxhyfwhy@163.com (X.W.); cindy0827@stu.xjtu.edu.cn (W.W.); drlin301@163.com (X.L.); chenxu_301@163.com (X.C.); zhumingxiang81@163.com (M.Z.); xuhongli@301hospital.com.cn (H.X.); 2 Medical School of Chinese PLA, Beijing 100853, China; 3 Department of Hepatobiliary Surgery, Chinese PLA 970th Hospital, Yantai 264001, China

**Keywords:** hepatocellular carcinoma, post-hepatectomy liver failure, inflammatory marker, net reclassification improvement, integrated discrimination improvement

## Abstract

Background: Post-hepatectomy liver failure (PHLF) remains a complication with the potential risk of mortality for hepatocellular carcinoma (HCC) patients. The systemic inflammatory response (SIR) has been demonstrated to be associated with a bad prognosis of liver cirrhosis and tumors. This study aims to evaluate the incremental prognostic value of inflammatory markers in predicting PHLF in patients with HCC. Methods: Clinical characteristics and variables were retrospectively collected in 2824 patients diagnosed with HCC who underwent radical hepatectomy from the First Medical Center of the General Hospital of the People’s Liberation Army. A recently published prognostic model for PHLF was used as the reference model. The increase in AUC (ΔAUC), integrated discrimination improvement (IDI), and the continuous version of the net reclassification improvement (NRI) were applied for quantifying the incremental value of adding the inflammatory markers to the reference model. A *p* value < 0.05 was considered statistically significant. Results: The reference PHLF model showed acceptable prediction performance in the current cohort, with an AUC of 0.7492 (95%CI, 0.7191–0.7794). The calculated ΔAUC associated with procalcitonin (PCT) was the only one that was statistically significant (*p* < 0.05), with a value of 0.0044, and demonstrated the largest magnitude of the increase in AUC. The continuous NRI value associated with the systemic immune-inflammation index (SII) was 35.79%, second only to GPS (46.07%). However, the inflammatory markers of the new models with statistically significant IDI only included WBC count, lymphocyte count, and SII. IDI associated with SII, meanwhile, was the maximum (0.0076), which was consistent with the performance of using the ΔAUC (0.0044) to assess the incremental value of each inflammatory variable. Conclusions: Among a wide range of inflammatory markers, only PCT and SII have potential incremental prognostic value for predicting PHLF in patients with radical resectable HCC.

## 1. Introduction

In contrast to many other cancer types, hepatocellular carcinoma (HCC) is growing rapidly worldwide, and it has ranked the sixth most common malignancy tumor and the second most lethal cancer globally [1,2,3]. Among all curable strategies, including liver transplantation (LT), radiofrequency ablation (RFA), and microwave ablation (MWA), surgical resection is still the primary radical therapeutic method for early-stage HCC in clinical practice [4,5]. With the continuous development of medical equipment and the persistent improvement in technical level, more patients with HCC, including BCLC B and C stages, can undergo surgical resection and achieve a good prognosis [6,7]. However, enlarged hepatectomy volume and long-term co-existing chronic liver diseases, such as fibrosis or cirrhosis, can increase the chances of post-hepatectomy liver failure (PHLF) [8]. Therefore, as a complication with a potential risk of mortality, PHLF remains a critically important concern for hepatic surgeons in preoperative evaluation.

Accurate prediction of PHLF in HCC patients before surgery is very essential for evaluating the feasibility and safety of liver resection. In recent decades, many studies have been conducted to solve this problem, and many PHLF prediction models based on preoperative variables have been established [9] To our knowledge, the widely used traditional clinical scoring systems of liver function have some shortcomings and limitations, such as the CP score (Child–Pugh) and MELD score (Model of End-Stage Liver Disease) [10,11,12]. Other models based on new preoperative noninvasive variables, including the albumin–bilirubin (ALBI) [13] and the albumin–indocyanine green evaluation (ALICE) scoring and grading system [14], have also been attempted to establish and proved to be reliable or superior in assessing PHLF of HCC patients undergoing hepatectomy.

Furthermore, infiltrating inflammatory cells are part of the tumor microenvironment, and a variety of inflammatory cytokines play a crucial role in the genesis and development of tumors [15,16]. Moreover, systemic inflammatory response (SIR) also plays a significant role in the pathogenesis and progression of liver cirrhosis, which has been demonstrated to be associated with a bad prognosis [17,18]. Based on the above facts, some studies began to explore the relationship between serum inflammatory markers and liver failure after hepatectomy. Recently, a new model was proposed, which was based on preoperative inflammatory indices and indocyanine green retention rate at 15 min (ICG R15) as a predictive tool for the liver failure of patients undergoing hepatectomy for HCC [19]. However, the model’s predictors did not include any inflammatory indices and the prognostic value of serum inflammatory markers was not thoroughly investigated and reported in the previous study.

The clinical inflammatory markers mainly included white blood cell (WBC) count, neutrophil count, lymphocyte count, monocyte count, C-reactive protein (CRP), interleukin-6 (IL-6), and procalcitonin (PCT). Moreover, many inflammation-based scores (IBSs) have been defined and evaluated for their relationship to disease prognosis in previous studies [20,21,22,23,24,25,26,27], including Glasgow prognostic score (GPS), modified Glasgow prognostic score (mGPS), neutrophil-to-lymphocyte ratio (NLR), platelet-to-lymphocyte ratio (PLR), lymphocyte-C-reactive protein ratio (LCR), lymphocyte-to-monocyte ratio (LMR), prognostic index (PI), prognostic nutritional index (PNI), systemic immune-inflammation index (SII), aspartate transaminase (AST)-to-neutrophil ratio index (ANRI), fibrinogen to albumin ratio (FAR), and albumin to fibrinogen ratio (AFR). This study aims to evaluate the incremental prognostic value of inflammatory markers in predicting PHLF in patients with HCC based on the established novel nomogram model.

## 2. Methods

### 2.1. Subjects

**Figure 1:**
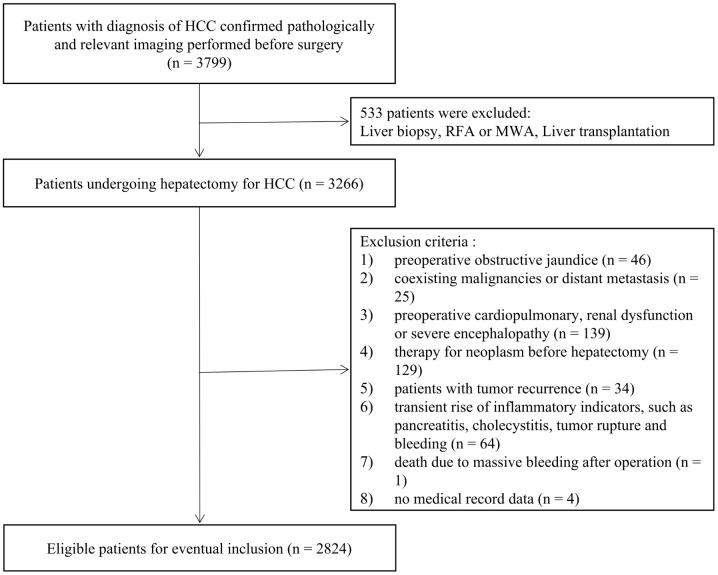
Flowchart of the patient enrolling process.

A total of 2824 HCC patients who underwent radical hepatectomy from the First Medical Center of the General Hospital of the People’s Liberation Army between January 2012 and December 2021 were finally enrolled in the present study. The study was approved by the Ethics Committee of the First Medical Center of the General Hospital of the People’s Liberation Army in compliance with the Declaration of Helsinki. Moreover, we have completed data desensitization of patients’ personal information in this study. The patient’s inclusion and exclusion criteria are consistent with those of the original model dataset. The enrollment flowchart of patients was presented in Figure 1.

Eligibility criteria included: (1) diagnosis of HCC confirmed by pathological examination; (2) no therapy for neoplasm before hepatectomy; (3) no coexisting malignancies; (4) no preoperative obstructive jaundice; and (5) no preoperative cardiopulmonary, renal dysfunction, severe encephalopathy. 

Exclusion criteria included: (1) patients with tumor recurrence or distant metastasis before hepatectomy; (2) patients who did not undergo relevant imaging (enhanced CT or MRI) within 1 month before surgery; (3) no data of liver function, coagulation function, and drainage volume of ascites on or after postoperative day 5.

### 2.2. Indocyanine Green Test

Before surgery, a dose of 50 mg indocyanine green dissolved in 10 mL of sterile water was injected through a peripheral vein (0.5 mg/kg). ICG-R15 was measured at 15 min after injection using a pulse spectrophotometer (DDG-3300 K, Nihon Kohden, Tokyo, Japan). Results were expressed as the percentage of ICG-R15 after injection.

### 2.3. Clinical Characteristics and Variables

All demographic characteristics were retrospectively aggregated from the electronic medical record, including age, sex, history of diabetes mellitus, hypertension, hepatitis B, and hepatitis C based on admission or discharge diagnosis. The amount of blood loss was obtained based on the surgical records. The tumor size (major nodule diameter) and determination of cirrhosis, as patients’ imaging data, were extracted based on the reporting of enhanced MRI first, and then enhanced CT. The result of ICG-R15 was recorded in a separate test report (the DDG test report of liver reserve function). Preoperative serum examination included WBC count, neutrophil count, lymphocyte count, monocyte count, platelet count, AST, albumin (ALB), prothrombin time (PT), fibrinogen (FIB), CRP, IL-6, and PCT. In addition, each of the IBSs was calculated and/or scored in this cohort study to evaluate its incremental value in predicting PHLF in HCC patients. The scoring details of IBSs were described in Table 1. 

### 2.4. Definitions of PHLF

Combined with data extraction and clinical practice, some patients would prolong their hospital stay due to wound healing, abdominal infection, or excessive drainage. Among these patients, the majority did not continue to recheck the status of liver function and/or coagulation function on or after postoperative day 5. Since PHLF was generally considered to be a deterioration of liver synthesis, excretion, and detoxification function after hepatectomy, it was determined in our study based on two international definition standards, including the recommendations of the International Study Group of Liver Surgery (ISGLS) [28] and the Memorial Sloan Kettering Cancer Centre (MSKCC) criteria [29]. The specific characteristics (on or after postoperative day 5) were described as follows: a total serum bilirubin value > 24 μmol/L and an international normalized ratio (INR) > 1.2, or serum bilirubin (SB) more than 70.1 µmol/L (4.1 mg/dL), or INR more than 2.5, or ascites drainage more than 500 mL/day.

Furthermore, patients discharged from the hospital within 5 days after surgery were considered to have no liver failure.

### 2.5. Reference Model

The reference model was recently published in a current journal within the relevant research topic [19]. It contained six predictors, including cirrhosis, PT, tumor size, ICG-R15%, blood loss, and AST-to-platelet ratio index (APRI), and an AUC of 0.845 in the derivation data. 

### 2.6. Statistical Analysis

We presented continuous variables as the median and interquartile range (IQR) and examined the differences among the groups using the Kruskal–Wallis. We presented categorical variables with the corresponding percentage and examined the differences using the ×2 or Fisher’s exact test. Associated inflammatory markers, which were particularly reported in previous literature, were added to the final predictive variables of the reference model [19]. For those inflammatory markers with a rightly skewed distribution, a natural logarithm was taken to convert them into a normal distribution. The percentages of missing data for all variables were calculated and presented (Supplementary Table S1). To avoid case deletion in analyses, the multiple imputation method was used to deal with the missing data in our data analysis. The change in the area under the ROC curve (AUC) (ΔAUC) between the model that included each inflammatory marker and the reference model was examined. We set the threshold as 10%, which is approximately the event rate in our data, for risk stratification to calculate sensitivity, specificity, accuracy, positive predictive value (PPV), and negative predictive value (NPV) rates, respectively. Furthermore, continuous net reclassification improvement (NRI) is considered a universal measure due to its ability to quantify the correct movement in categories, and integrated discrimination improvement (IDI) is a useful indicator for evaluating new predictive factors as it integrates all changes in the predicted value of risk [30,31,32,33]. To further evaluate the incremental value of each inflammatory marker, continuous NRI and IDI were also calculated to compensate for the deficiency of AUC. The above statistical results were obtained by using the predictABEL package in R software. We determined the 95% confidence intervals (CIs) through bootstrapping 1000 samples. A *p* value < 0.05 was considered statistically significant. All statistical analyses were conducted using the IBM SPSS 23.0 and R software (version 3.6.1).

## 3. Results

### 3.1. Descriptive Data

All 2824 patients (2405 men, 85.1%; mean age 55.77 ± 10.61 years) who met the eligibility criteria were enrolled, including 268 patients (242 men, 90.2%; mean age 56.70 ± 10.01 years) with PHLF events. The baseline characteristics of HCC patients with and without PHLF events were shown in Table 2. There were no statistically significant differences between both groups regarding age, sex, hepatitis (HBsAg or HCV), and underlying diseases, including diabetes mellitus and hypertension. 

In this retrospective study, the final variable data of the reference model were statistically analyzed, including cirrhosis (39.7%), tumor size (5.68 ± 3.45), blood loss (364.75 ± 503.13), ICG-R15 (5.55 ± 4.83), preoperative PT (13.67 ± 0.98), and APRI (0.58 ± 0.55). The values of all six variables were increased in the subgroup of patients with PHLF, especially liver cirrhosis (55.5%), tumor size (7.03 ± 3.96), blood loss (595.45 ± 814.65), and APRI (0.90 ± 0.79). Inflammation-related variables refer to inflammatory makers, including white cell (5.68 ± 1.82), neutrophil (3.34 ± 1.47), lymphocyte (1.75 ± 0.63), monocyte (0.42 ± 0.18), CRP (6.97 ± 15.73), IL-6 (12.48 ± 26.48), and PCT (0.12 ± 0.31), and IBSs, including GPS (0, 80.2%), mGPS (0, 80.4%), PI (0, 84.0%), PNI (1, 18.6%), SII (0, 53.0%), LCR (0, 59.9%), NLR (0, 96.8%), LMR (1, 82.4%), PLR (0, 86.0%), ANRI (11.56 ± 10.49), FAR (0.08 ± 0.04), and AFR (14.48 ± 4.21). Most of the inflammatory markers did not differ significantly from the subgroup of patients with PHLF, except for lymphocyte (1.51 ± 0.56), PCT (0.28 ± 0.66), PNI (1, 35.2%), LCR (0, 46.2%), LMR (1, 73.4%), and ANRI (16.86 ± 15.82). Data statistics for all variables were also presented in Table 2.

### 3.2. Assessment of the Incremental Value of Inflammation-Related Variables over the Original Model

The reference PHLF model was restored and showed acceptable prediction performance in the current cohort, with an AUC of 0.7492 (95% CI, 0.7191–0.7794), indicating a significant decrease in prognostic power compared to the previous study. The model that included each inflammatory marker demonstrated different discrimination compared to the reference model. Variables that improve the model discrimination, namely the positive ΔAUC, included lymphocyte count (0.0031), monocyte count (0.0004), CRP (0.0017), IL-6 (0.0001), PCT (0.0044), GPS (0.0001), mGPS (0.0013), PI (0.0005), SII (0.0044), ANRI (0.0004), LMR (0.0003), PLR (0.0005), FAR (0.0033), and AFR (0.0035). Conversely, variables with a negative ΔAUC were neutrophil (−0.0006), PNI (−0.0004), LCR (−0.0001), and NLR (−0.0001). However, the ΔAUC associated with PCT was the only one that was statistically significant (*p* < 0.05) and demonstrated the largest magnitude of the increase in AUC. Additionally, the results of sensitivity, specificity, accuracy, PPV, and NPV rates based on the specific classification threshold for PHLF were presented in Supplementary Table S2.

Furthermore, continuous NRI and IDI were examined as new measures to evaluate the improvement in the field of risk assessment. Continuous NRI associated with inflammatory biochemical markers ranged from −5.19% (monocyte count) to 16.26% (neutrophil count), while the continuous NRI associated with IBSs ranged from 5.78% (PLR) to 46.07% (GPS). Most of the continuous NRI was statistically significant (*p* < 0.05), except for those attained by including the variable of monocyte count, CRP, IL-6, PCT, or PLR in the reference model. The continuous NRI level was greater than 20% in models by the addition of ANRI (21.51%), LCR (23.09%), NLR (31.63%), SII (35.79%), and GPS (46.07%). However, inflammatory markers of the new models with statistically significant IDI only included WBC count (0.0041), lymphocyte count (0.0034), and SII (0.0076). Table 3 reported the results of all the measures used to evaluate the incremental value of an inflammatory marker for predicting PHLF.

## 4. Discussion

Although the level of medical technology continues to improve and progress, PHLF is still one of the main clinical fatal complications. Over the past few decades, due to the clinical defects of traditional liver function evaluation indexes, easily accessible and effective predictive indexes related to PHLF have been constantly explored. Recently, inflammatory markers, as the objective reflection of inflammation level, have been demonstrated as significant predictive variables to influence the prognostic risk prediction of solid tumors, including HCC [23,27], lung cancer [24], nasopharyngeal carcinoma [25], and pancreatic cancer [22]. To explore and validate a preoperative potential prognostic factor for patients with HCC, we performed a statistical analysis of all inflammatory markers in a relatively large sample (2824 patients). In the present study, we showed the incremental prognostic value of each inflammatory marker over an established PHLF nomogram based on easily available predictors in HCC patients.

With the extensive development of research on new biomarkers, the methodology for evaluating their predictive incremental performance has also been rapidly developing and constantly improving. Therefore, appropriate statistical measures are necessary to be taken in the process of evaluating incremental value analysis. The inflammatory marker, as a new preoperatively available predictor, is intended to improve the accuracy of the predictive model for PHLF so that clinicians can make reasonable treatment choices. To obtain meaningful conclusions about the clinical usefulness of new predictors, we focus on three measures of improvement in model performance: an increase in the AUC, the IDI, and the continuous version of the NRI.

The new model is a nested relationship model based on the reference nomogram with a new inflammatory predictor. In the process of establishing the reference model, only AUC was used to report and evaluate the effectiveness of the model, and the AUC, or its empirical estimator, often called the c-statistic, was generally relied upon as the primary measure of improved explanatory ability in the field of risk assessment. Therefore, we first evaluated the value of AUC for each new model and observed the maximal increase in the AUC from 0.7492 to 0.7536 by adding PCT to the reference model, which was the only one with statistical significance (*p* < 0.05). The calculated ΔAUC ranged from −0.0006 to 0.0044, without noticeable improvement in model performance. Since the AUC is an overall measure of differentiation, it reflects too general information and some apparent improvements in sensitivity for a specific classification threshold do not translate into improvements in the AUC. In addition, some studies have emphasized the limitations of the AUC [34,35,36], especially the inadequate interpretation of changes in this statistic. For example, the AUC value of the new model is roughly the same as that of the old model, but the predictive ability of the two models in different regions is different, so the incremental values of inflammatory markers cannot be judged solely on the size of the ΔAUC.

The continuous NRI, as a measure focused on reclassification tables, quantifies the correct movement in categories without consideration for any risk categorization [37]. The positive continuous NRI, which is based on qualitative counting judgment, represents the increase in the risk predictive value of the new model in the event group and the decrease in the non-event group. According to the calculation of continuous NRI, the probability of correct risk classification of the new model with the addition of variable GPS or SII has been significantly improved, although the value of AUC has no statistically significant increase. Furthermore, the IDI is another index that is not dependent on risk classification but directly dependent on event probability and is an indicator that combines all the changes in the predicted value of risk. Since IDI takes into account the situation of different cut-off values, it can be used to reflect the overall improvement of the model, and, to some extent, it can make up for the shortcomings of NRI and the defects of AUC. Of all inflammatory markers, the IDI associated with SII was the largest, which was consistent with the performance of using the ΔAUC to assess the incremental value of each inflammatory marker.

Based on the comparison of statistical analysis results, PCT and SII have probably demonstrated incremental value in predicting PHLF in HCC patients. The release of elevated amounts of PCT in the circulation during infection and inflammation has been described and confirmed approximately 20 years ago [38]. As a very sensitive inflammatory biochemical marker, PCT has become an innovative diagnostic indicator for early diagnosis of infection, treatment monitoring, and prognosis judgment. For example, a patient in our dataset with PHLF had a prediction probability of 7.45% in the original model, while the prediction probability was 14.77% with the addition of procalcitonin (1.05 ng/mL, higher than 99% of all patients), which showed a significant improvement in prediction and will change the clinical decision. SII, as a stable indicator reflecting the inflammatory status of the whole human body, was a new index developed and proposed based on the prognostic scores of HCC patients with a high risk of recurrence and death after radical resection [23]. Then, SII has been confirmed by several studies to have poor prognostic values in several diseases and could be a promising tool for decision making on cancer treatment strategies.

As previous studies have demonstrated, high levels of serum inflammatory markers are strongly associated with poor prognosis in cirrhosis and solid tumors. Cirrhosis is an important indicator for the preoperative evaluation of liver function and surgical feasibility and has been used as the final predictor by several PHLF prediction models, including reference nomogram [19,39,40]. Thus, it is not surprising that PCT and SII can improve the model’s predictive ability of PHLF in patients with HCC.

## 5. Limitations

There are several limitations to the present study that are worth discussing. First, although our study has a relatively large sample size, it is still a single-center cohort study. Then, it is also a cohort study with a retrospective nature. Generally, some indicators, such as ICG-R15%, CRP, PCT, and CRP, are not used as routine preoperative test items, which will result in missing data. A future multicenter prospective study is needed to confirm our findings. Third, as mentioned above, among more than a dozen definitions of PHLF that have been applied [9], we choose two definitions to determine PHLF. Although this will have a certain impact on the research results, it is more likely to improve the accuracy of the study and reduce the risk bias of the model than excluding a large number of the study population. Finally, further studies are needed to investigate the relationship between the significant changes in inflammatory markers due to acute inflammation, such as pancreatitis and cholecystitis, and PHLF in HCC patients.

## 6. Conclusions

In this paper, we have applied three methods for quantifying improvement in model performance resulting from the addition of each new inflammatory marker. Despite the contradiction between the results of different methodologies, this study provides an assessment of incremental value to be guided by a risk prediction ability to improve clinical practice. Given the results above, the novel nomogram with the addition of PCT or SII showed superior prediction of PHLF in patients with resectable HCC than the reference model.

## Figures and Tables

**Table 1:** Abbreviation and definition of each inflammation-based scoring system.

Abbreviation	Definition of Each Scoring System	Score
GPS: Glasgow prognostic score		
	CRP (<10 mg/L) and albumin (≥35 g/L)	0
	CRP (≥10 mg/L) or albumin (<35 g/L)	1
	CRP (≥10 mg/L) and albumin (<35 g/L)	2
mGPS: modified Glasgow prognostic score		
	CRP (≤10 mg/L) and albumin (≥35 g/L)	0
	CRP (≤10 mg/L) and albumin (<35 g/L)	0
	CRP (>10 mg/L) and albumin (≥35 g/L)	1
	CRP (>10 mg/L) and albumin (<35 g/L)	2
PI: prognostic index		
	CRP (≤10 mg/L) and white blood cell count (≤11 × 10^9^/L)	0
	CRP (≤10 mg/L) and white blood cell count (>11 × 10^9^/L)	1
	CRP (>10 mg/L) and white blood cell count (≤11 × 10^9^/L)	1
	CRP (>10 mg/L) and white blood cell count (>11 × 10^9^/L)	2
PNI: prognostic nutritional index		
	Albumin (g/L) + 5 × total lymphocyte count (10^9^/L) ≥ 45	0
	Albumin (g/L) + 5 × total lymphocyte count (10^9^/L) < 45	1
SII: systemic immune-inflammation index		
	Platelet count (×10^9^/L) × neutrophil count (×10^9^/L)/ lymphocyte count (×10^9^/L) < 305	0
	Platelet count (×10^9^/L) × neutrophil count (×10^9^/L)/ lymphocyte count (×10^9^/L) ≥ 305	1
LCR: lymphocyte-C-reactive protein ratio		
	10^4^ × lymphocyte count (10^9^/L): CRP (mg/L) > 6000	0
	10^4^ × lymphocyte count (10^9^/L): CRP (mg/L) ≤ 6000	1
NLR: neutrophil-to-lymphocyte ratio		
	Neutrophil count(10^9^/L): lymphocyte count(10^9^/L) < 5:1	0
	Neutrophil count(10^9^/L): lymphocyte count(10^9^/L) ≥ 5:1	1
LMR: lymphocyte-to-monocyte ratio		
	Lymphocyte count (10^9^/L): monocyte count (10^9^/L) < 3	0
	Lymphocyte count (10^9^/L): monocyte count (10^9^/L) ≥ 3	1
PLR: platelet-to-lymphocyte ratio		
	Platelet count(10^9^/L): lymphocyte count(10^9^/L) < 150:1	0
	Platelet count(10^9^/L): lymphocyte count(10^9^/L) ≥ 150:1	1
	Platelet count(10^9^/L): lymphocyte count(10^9^/L) > 300:1	2

**Table 2:** Characteristics of patients in our study cohort.

Variable	All Patients (*n* = 2824)	Clinical Classifcation on Outcome		
		Patients without PHLF (*n* = 2556)	Patients with PHLF (*n* = 268)	*p* Value
Demographic characteristics				
Age (years)	56 (49–64)	56 (49–63)	58 (50–64)	0.017
Sex				
Male	2405 (85.1%)	2163 (84.6%)	242 (90.2%)	
Female	419 (14.8%)	393 (15.3%)	26 (9.7%)	
HBsAg				0.018
Positive	1966 (69.6%)	1770 (69.2%)	196 (73.1%)	
Negative	858 (30.4%)	786 (30.8%)	72 (26.9%)	
HCV				0.268
Positive	107 (3.8%)	101 (3.9%)	6 (2.2%)	
Negative	2717 (96.2%)	2455 (96.1%)	262 (97.8%)	
Diabetes mellitus				0.646
Yes	616 (21.8%)	561 (21.9%)	55 (20.5%)	
No	2208 (78.1%)	1995 (78.0%)	213 (79.4%)	
Hypertension				0.003
Yes	758 (26.8%)	707 (27.6%)	51 (19.0%)	
No	2066 (73.1%)	1849 (72.3%)	217 (80.9%)	
Final variables of the original model				
Cirrhosis				<0.001
Yes	1123 (39.7%)	974 (38.1%)	149 (55.5%)	
No	1701 (60.2%)	1582 (61.8%)	119 (44.4%)	
Tumor size (cm)	4.8 (3.1–7.4)	4.6 (3.0–7.3)	6.2 (3.95–9.55)	0.001
Blood loss (ml)				<0.001
<400	1916 (68.1%)	1786 (70.1%)	130 (48.7%)	
≥400	899 (31.9%)	762 (29.9%)	137 (51.3%)	
ICG-R15 (%)	4.5 (2.9–6.9)	4.4 (2.9–6.7)	6.1 (3.8–8.6)	<0.001
PT (s)	13.6 (13.1–14.2)	13.6 (13.0–14.2)	14.1 (13.4–14.8)	<0.001
APRI	0.41 (0.28–0.67)	0.40 (0.27–0.64)	0.62 (0.39–1.12)	<0.001
Inflammation-related variables				
White cell (10^9^/L)	5.47 (4.48–6.59)	5.51 (4.52–6.62)	4.98 (3.84–6.27)	0.001
Neutrophil (10^9^/L)	3.05 (2.38–3.96)	3.06 (2.41–3.97)	2.89 (2.11–3.90)	0.271
Lymphocyte (10^9^/L)	1.67 (1.31–2.09)	1.69 (1.33–2.12)	1.44 (1.09–1.86)	0.523
Monocyte (10^9^/L)	0.39 (0.31–0.50)	0.39 (0.31–0.49)	0.38 (0.29–0.50)	0.029
Platelet (10^9^/L)	163 (127–207)	164 (129–208)	142 (96.5–186.5)	<0.001
AST (U/L)	26.1 (19.6–37.3)	25.3 (19.4–36.1)	33.8 (24.6–48.9)	<0.001
ALB (g/L)	40.6 (38.3–43.0)	40.7 (38.4–43.1)	39.4 (36.8–41.8)	<0.001
FIB (g/L)	2.81 (2.40–3.41)	2.82 (2.40–3.40)	2.72 (2.29–3.42)	0.315
CRP (mg/L)	1.9 (0.9–5.2)	1.7 (0.9–5.1)	3.17 (1.0–6.4)	0.144
IL-6 (pg/mL)	4.68 (2.98–10.94)	4.55 (2.91–11.10)	6.2 (3.44–10.20)	0.184
Procalcitonin (ng/mL)	0.05 (0.03–0.08)	0.05 (0.03–0.08)	0.13 (0.04–0.24)	<0.001
GPS				0.039
0	879 (80.2%)	793 (81.2%)	86 (72.3%)	
1	209 (19.1%)	176 (18.0%)	33 (27.7%)	
2	8 (0.7%)	8 (0.8%)	0	
mGPS				0.876
0	931 (85.0%)	831 (85.1%)	100 (84.1%)	
1	130 (11.9%)	115 (11.8%)	15 (12.6%)	
2	35 (3.2%)	31 (3.2%)	4 (3.4%)	
PI				0.558
0	921 (84.0%)	823 (84.2%)	98 (82.4%)	
1	169 (15.4%)	149 (15.2%)	20 (16.8%)	
2	6 (0.5%)	5 (0.5%)	1 (0.8%)	
PNI				0.341
0	2289 (81.4%)	2116 (83.1%)	173 (64.8%)	
1	524 (18.6%)	430 (16.9%)	94 (35.2%)	
SII				<0.001
0	1492 (53.0%)	1343 (52.7%)	149 (55.8%)	
1	1321 (47.0%)	1203 (47.3%)	118 (44.2%)	
LCR				0.002
0	655 (59.9%)	600 (61.5%)	55 (46.2%)	
1	439 (40.1%)	375 (38.5%)	64 (53.8%)	
NLR				0.004
0	2723 (96.8%)	2468 (97.0%)	255 (95.5%)	
1	90 (3.2%)	78 (3.1%)	12 (4.5%)	
LMR				0.003
0	494 (17.6%)	423 (16.6%)	71 (26.6%)	
1	2319 (82.4%)	2123 (83.4%)	196 (73.4%)	
PLR				0.436
0	2418 (86.0%)	2193 (86.1%)	225 (84.3%)	
1	371 (13.2%)	331 (13.0%)	40 (15.0%)	
2	24 (0.9%)	22 (0.9%)	2 (0.7%)	
ANRI	8.60 (5.83–13.75)	8.32 (5.67–13.05)	12.07 (7.84–19.71)	0.454
FAR	0.07 (0.06–0.09)	0.07 (0.06–0.09)	0.07 (0.06–0.09)	0.399
AFR	14.42 (11.63–17.08)	14.44 (11.73–17.04)	14.33 (11.28–17.31)	0.399

Abbreviations: HBsAg, hepatitis B surface antigen; HCV, hepatitis C virus; ICG-R15, indocyanine green retention rate at 15 min; PT, prothrombin time; APRI, aspartate transaminase-to-platelet ratio index; AST, aspartate transaminase; ALB, albumin; FIB, fibrinogen; CRP, C-reactive protein; IL-6, interleukin-6; GPS, Glasgow prognostic score; mGPS, modified Glasgow prognostic score; PI, prognostic index; PNI, prognostic nutritional index; SII, systemic immune-inflammation index; LCR, lymphocyte-C-reactive protein ratio; NLR, neutrophil-to-lymphocyte ratio; LMR, lymphocyte-to-monocyte ratio; PLR, platelet-to-lymphocyte ratio; ANRI, aspartate transaminase-to-neutrophil ratio index; FAR, fibrinogen to albumin ratio; AFR, albumin to fibrinogen ratio.

**Table 3:** Assessment of incremental value of predicted probability of PHLF with the model that included the inflammatory variables compared with the baseline model.

Variable	AUC (95%CI)	ΔAUC	*p* Value	Continuous NRI (95%CI)	*p* Value	IDI (95%CI)	*p* Value
WBC	0.7492 (0.7189–0.7795)	0	0.9964	0.1569 (0.0317–0.2821)	0.0140	0.0041 (0.0007–0.0076)	0.0182
Neutrophil	0.7486 (0.7184–0.7788)	−0.0006	0.8183	0.1626 (0.0383–0.2869)	0.0130	0.0024 (0.0001–0.0050)	0.0633
Lymphocyte	0.7523 (0.7221–0.7822)	0.0031	0.3849	0.1558 (0.0309–0.2807)	0.0145	0.0034 (0.0006–0.0062)	0.0186
Monocyte	0.7496 (0.7195–0.7797)	0.0004	0.3979	−0.0519 (−0.1762–0.0725)	0.4137	0.0000 (−0.0004–0.0003)	0.8437
CRP	0.7509 (0.721–0.7808)	0.0017	0.2961	0.0213 (−0.1030–0.1457)	0.7365	0.0005 (−0.0011–0.0021)	0.5612
IL-6	0.7493 (0.7191–0.7795)	0.0001	0.7819	0.0175 (−0.1049–0.1399)	0.7795	0.0000 (−0.0003–0.0004)	0.7797
PCT	0.7536 (0.7236–0.7837)	0.0044	0.0265	0.0996 (−0.0249–0.2241)	0.1167	0.0033 (−0.0010–0.0076)	0.1319
GPS	0.7493 (0.7192–0.7795)	0.0001	0.8890	0.4607 (0.3549–0.5666)	<0.0001	0.0005 (−0.0010–0.0020)	0.5350
mGPS	0.7505 (0.7205–0.7804)	0.0013	0.5532	0.1654 (0.0540–0.2769)	0.0036	0.0017 (−0.0005–0.0040)	0.1327
PI	0.7497 (0.7197–0.7798)	0.0005	0.7979	0.0902 (−0.0160–0.1963)	0.0960	0.0017 (−0.0002–0.0037)	0.0796
PNI	0.7488 (0.7184–0.7792)	−0.0004	0.7172	0.1500 (0.0289–0.2711)	0.0152	0.0012 (0.0000–0.0024)	0.0582
SII	0.7536 (0.7236–0.7836)	0.0044	0.3593	0.3579 (0.2343–0.4816)	<0.0001	0.0076 (0.0030–0.0121)	0.0011
LCR	0.7491 (0.7189–0.7792)	−0.0001	0.7071	0.2309 (0.1060–0.3558)	0.0003	0.0001 (−0.0002–0.0004)	0.4133
NLR	0.7491 (0.7188–0.7794)	−0.0001	0.9030	0.3163 (0.2190–0.4137)	<0.0001	0.0005 (−0.0007–0.0017)	0.4470
ANRI	0.7496 (0.7197–0.7795)	0.0004	0.8767	0.2151 (0.0898–0.3405)	0.0008	0.0020 (−0.0006–0.0045)	0.1407
LMR	0.7495 (0.7193–0.7796)	0.0003	0.7183	0.1544 (0.0416–0.2673)	0.0073	0.0003 (−0.0005–0.0011)	0.4363
PLR	0.7497 (0.7196–0.7799)	0.0005	0.7353	0.0578 (−0.0592–0.1748)	0.3330	0.0009 (−0.0010–0.0028)	0.3462
FAR	0.7525 (0.7226–0.7823)	0.0033	0.2264	0.1786 (0.0565–0.3007)	0.0041	0.0013 (−0.0014–0.0041)	0.3412
AFR	0.7527 (0.7229–0.7825)	0.0035	0.1972	0.1621 (0.0374–0.2868)	0.0108	0.0010 (−0.0015–0.0036)	0.4292

## Data Availability

The original contributions presented in the study are included in the article/Supplementary Materials; further inquiries can be directed to the corresponding author/s.

## Supplementary Materials

The following supporting information can be downloaded at: https://www.mdpi.com/article/10.3390/life13101990/s1, Table S1: The number and percentage of missing values for each variable; Table S2: The predictive ability of each inflammatory marker added to the reference model for PHLF.
